# Supplementary material for: CRISPR-free RNA base editing mediated PTC-readthrough restores hearing in mice with Otof nonsense mutation
Source: Nat Commun. 2025 Dec 6;17:413. doi: 10.1038/s41467-025-67112-w (PMC12796263; doi:10.1038/s41467-025-67112-w)
Supplement: Supplementary file 1 — Supplementary Information [file 41467_2025_67112_MOESM1_ESM.pdf]

CRISPR-free RNA Base Editing Mediated PTC-readthrough Restores Hearing in Mice with *Otof* Nonsense Mutation

Hanxiao Sun[1,#], Qi Teng[2,#], Wenqing Liu[3,4,#], Rui Guo[2,#], Menghua Li[2,#], Wei Xiong[2], Qiang Huang[1,] Qianru Yu[2], Nan Luo[1], Yang Li[2,5], Jinghui Song[1], Shusheng Gong[2,6] , Xi Shi[7,*], Chengqi Yi[1,8,9,10,*], Ke Liu[2,5,6,*]

[1]The National Key Laboratory of Gene Function Studies and Manipulation, School of Life Sciences, Peking University, Beijing, 100871, China.

[2]Department of Otolaryngology Head and Neck Surgery, Beijing Friendship Hospital, Capital Medical University; Beijing 100050, China.

[3]School of Life Sciences, Tsinghua University; Beijing, China.

[4]Tsinghua-Peking Joint Center for Life Sciences, Tsinghua University; Beijing, China.

[5]Beijing Clinical Research Institute; Beijing 100050, China.

[6]Clinical Center for Hearing Loss, Capital Medical University; Beijing 100050, China.

[7]Academician Workstation of Hainan University (Sanya), School of Pharmaceutical Sciences, Hainan University; Haikou, Hainan 570228, China.

[8]Peking-Tsinghua Center for Life Sciences, Peking University; Beijing, China.

[9]Department of Chemical Biology and Synthetic and Functional Biomolecules Center, College of Chemistry and Molecular Engineering, Peking University; Beijing 100871, China.

[10]Beijing Advanced Center of RNA Biology (BEACON), Peking University; Beijing, China.

[#]These authors contributed equally to this work.

*Corresponding author. Email: shixi2023@hainanu.edu.cn (X.S,), chengqi.yi@pku.edu.cn (C.Y.), liuke@ccmu.edu.cn (K.L.)

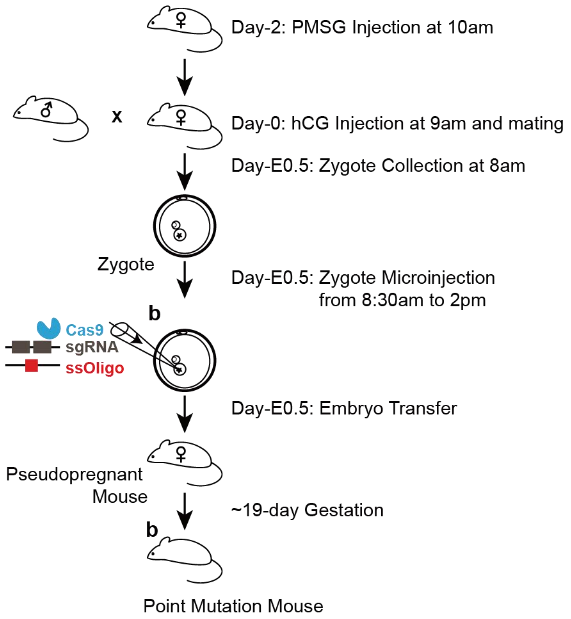

Supplementary Figure 1 | A Schematic diagram showing the step of the construction of *Otof c.1315C>T* (p.R439*) mouse model.

The procedure begins with superovulation (pregnant mare serum gonadotrophin (PMSG) injection) and is followed by mating after hCG administration. Zygotes are collected on embryonic day 0.5 (E0.5) and undergo pronuclear microinjection of CRISPR/Cas9 components—including Cas9 protein, guide RNA (sgRNA), and a donor oligonucleotide—within a critical window post-collection. Successfully injected embryos are transferred to pseudopregnant surrogate females, culminating in the birth of potential founder mice carrying the intended point mutation after a 19-day gestation.

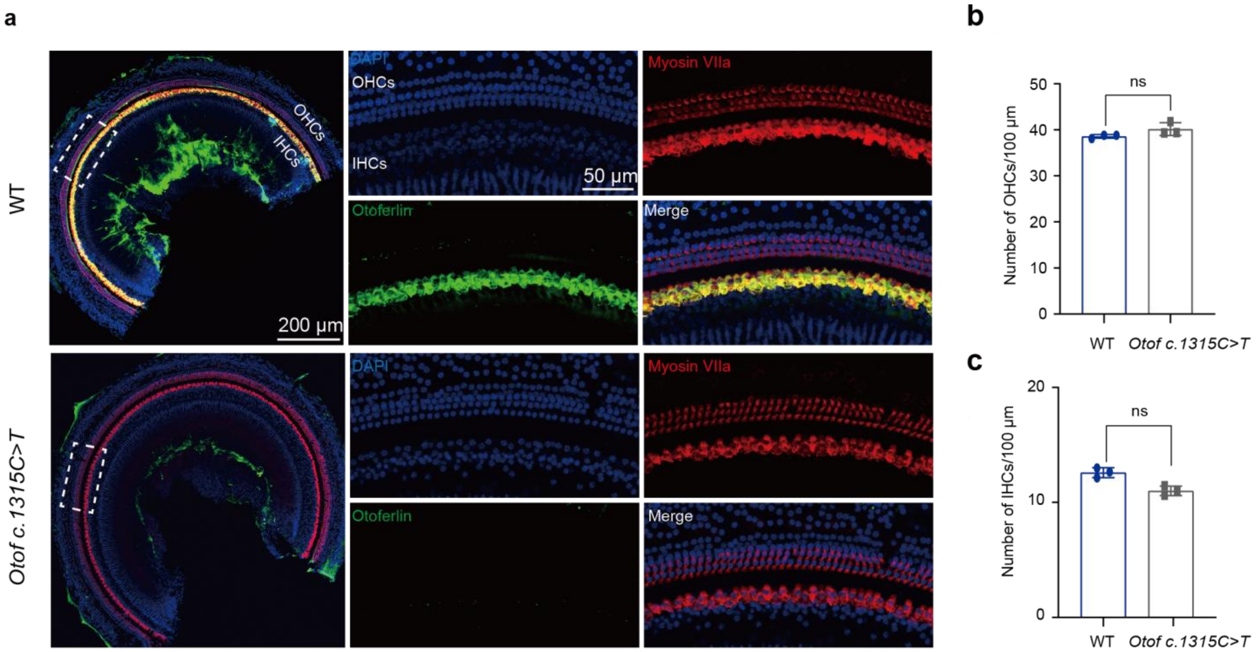

Supplementary Figure 2 | Immunostaining evaluation of HCs of *Otof c.1315C>T* (p.R439*) mice.

(a) The whole mount staining shows no differences in the number of HCs in *Otof c.1315C>T* (p.R439*) mice and WT mice. The experiment was repeated three times, and yielded consistent results across all replicates. The white frames indicate the enlarged regions. Myosin Ⅶa (in red), otoferlin (in green), DAPI (in blue). **(b-c)** Quantification of numbers of OHCs and IHCs of WT (n = 3 biological samples) and *Otof c.1315C>T* (p.R439*) mice (n = 3 biological samples). Data are represented as the mean ± SD. Data was analyzed by paired t-test. OHCs: outer hair cells, P = 0.1452 **(b)**; IHCs: inner hair cells, P = 0.9750 **(c)**. Scale Bars, 200 μm or 50 μm.

a

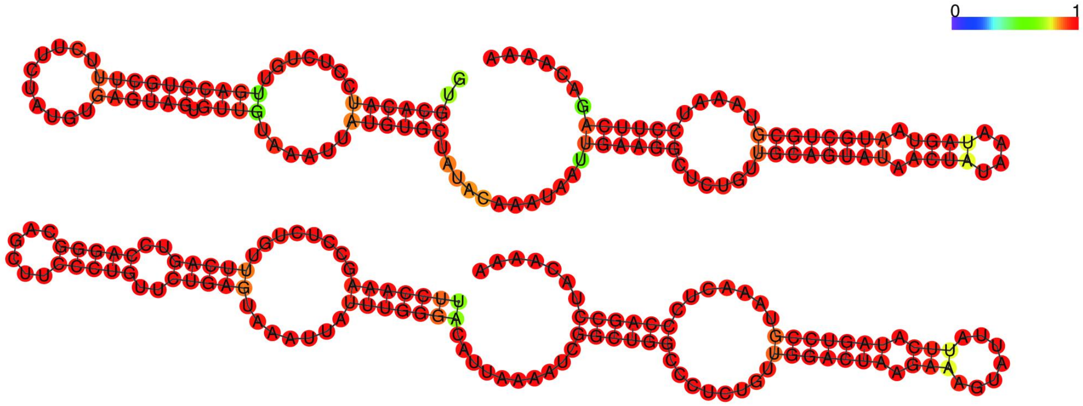

b

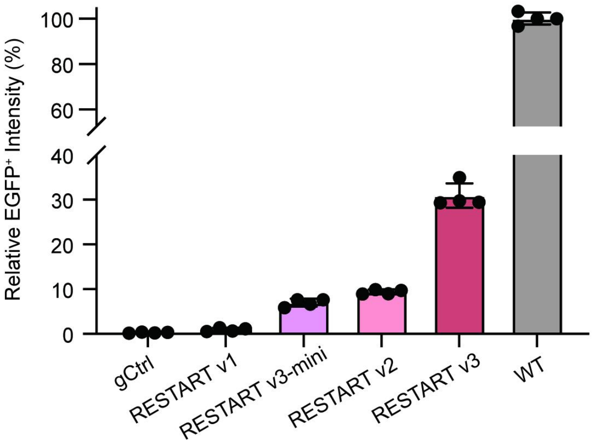

Supplementary Figure 3 | RESTART v3 can improve the efficiency of *Otof* premature termination codon (PTC) readthrough. (a) Predicted secondary structure of the gACA19 and gACA36 scaffolds. Red and violet colors represent high and low probabilities, respectively. Structures are predicted using RNAfold. **(b)** The indicated gACA19-PTC-reporter, DKC1 iso3, and tRNA-R-TCT-1-1 were transfected into HEK293T cells. Bar plot showing the relative fraction of EGFP intensity. n = 4 biological replicates. Data are represented as the mean ± SD.

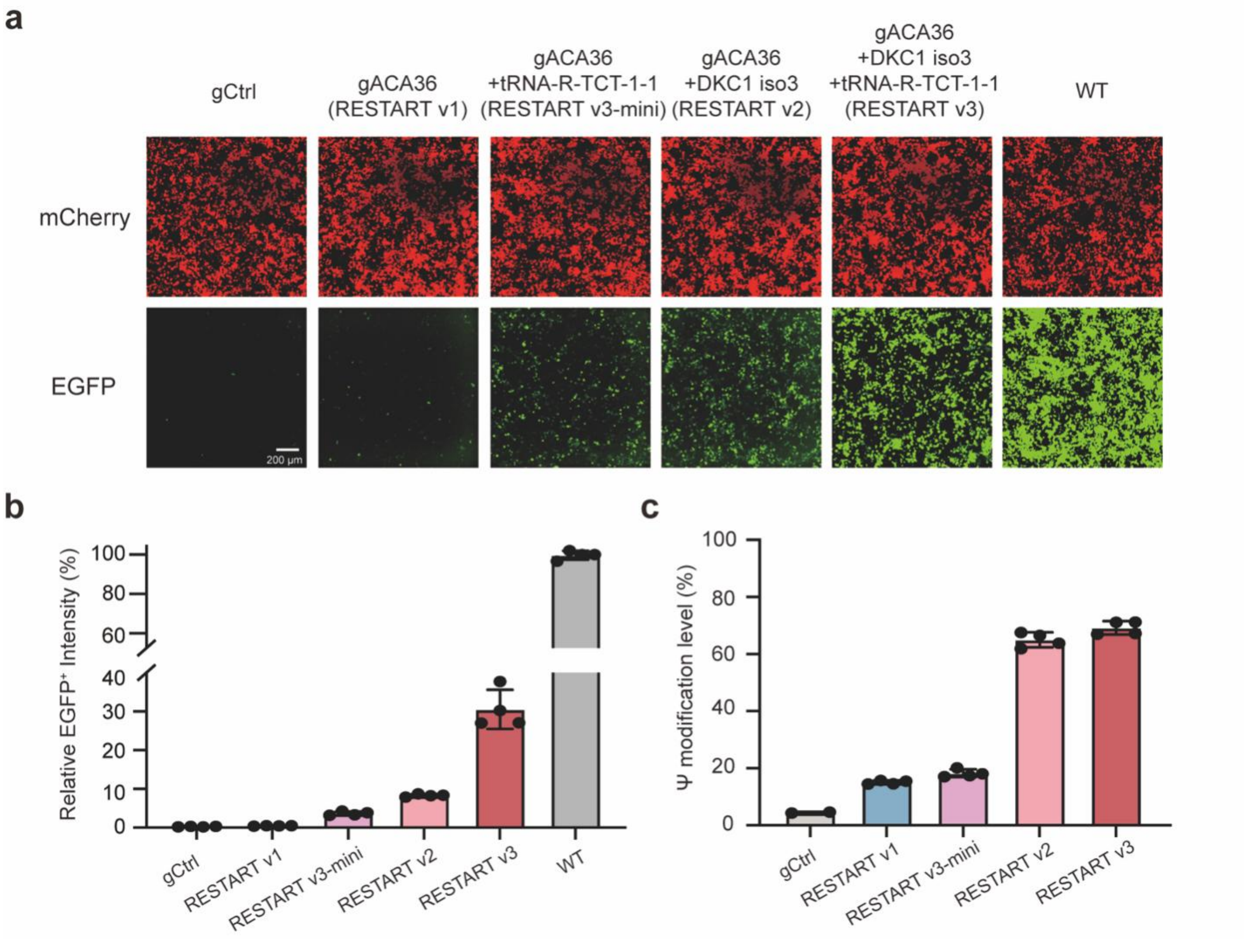

Supplementary Figure 4 | gACA36 has a comparable performance to gACA19.

(a-c) The gACA36-PTC-reporter, DKC1 iso3, and tRNA-R-TCT-1-1 were transfected into HEK293T cells. Representative fluorescence images of cells, and the experiment was repeated four times, and yielded consistent results across all replicates **(a)**. Bar plot showing the relative fraction of EGFP intensity **(b)**. Bar plot showing different modification level across different RESTART versions **(c)**. n = 4 biological replicates. Data are represented as the mean ± SD. Scale Bars, 200 μm.

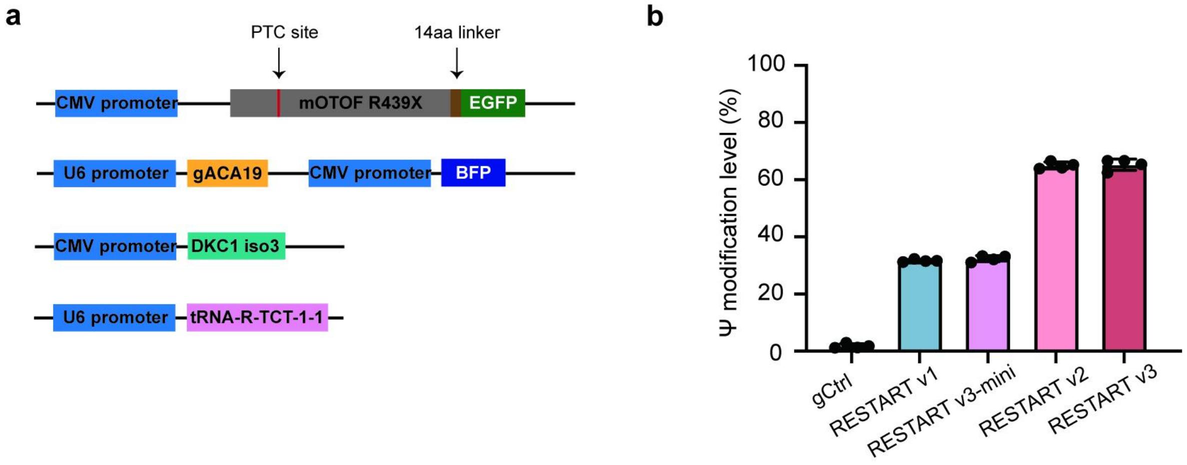

Supplementary Figure 5 | RESTART v3 incorporates pseudouridine modification in PTC site of full-length *Otof* mRNA.

(a) The schematics of mOTOF (Full length, R439X)-PTC-reporter, gACA19, DKC1-iso3 and tRNA-R-TCT-1-1 constructs. **(b)** The indicated mOTOF (Full length, R439X)-PTC-reporter, gACA19, DKC1-iso3 and tRNA-R-TCT-1-1 were transfected into HEK293T cells. Bar plot showing different modification level across different RESTART versions. n = 4 biological replicates. Data are represented as the mean ± SD.

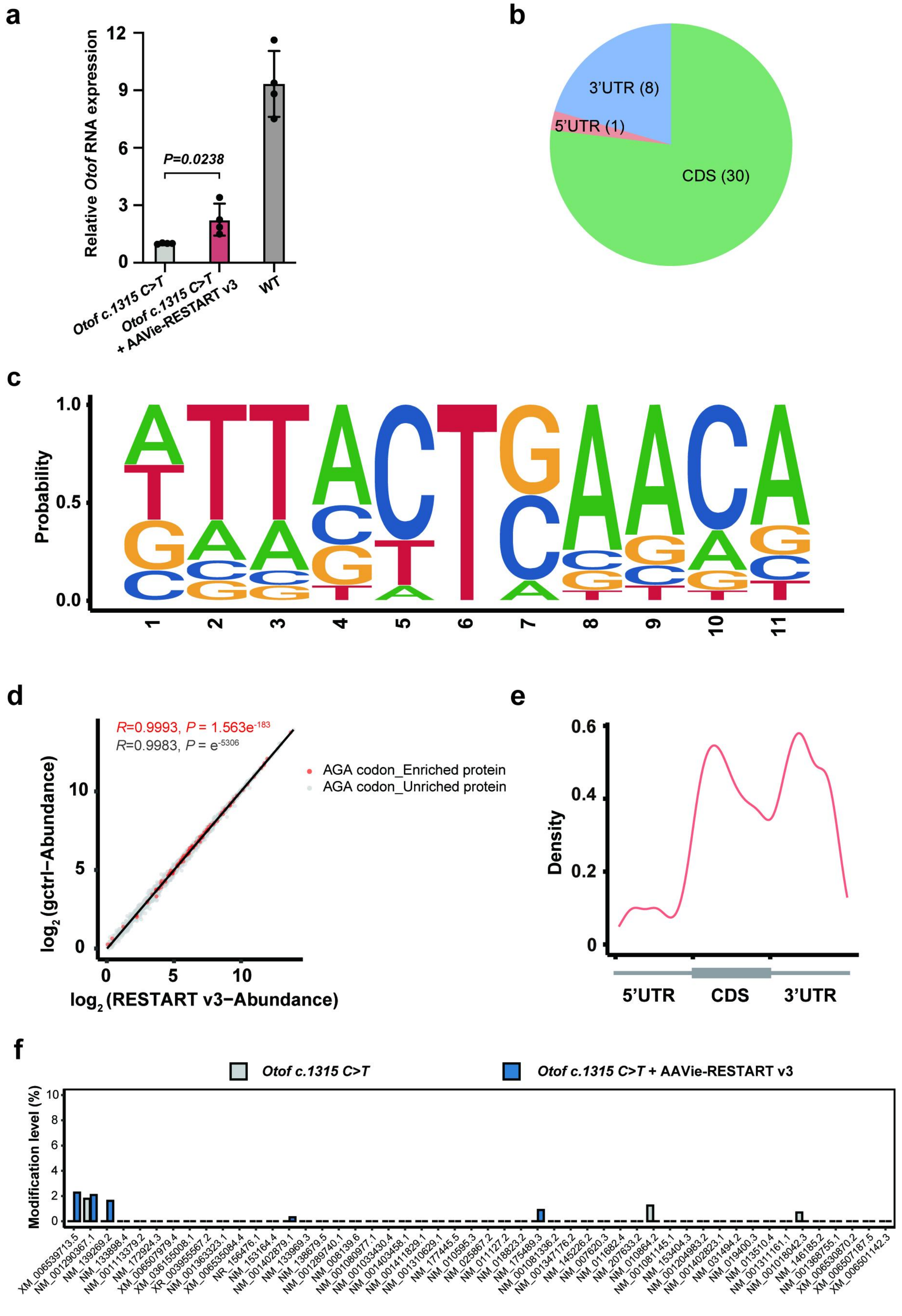

a
Relative Otof RNA expression
12
9
6
3
0
P=0.0238
Otof c.1315 C>T
Otof c.1315 C>T + AAVie-RESTART v3
WT
b
3'UTR (8)
5'UTR (1)
CDS (30)
c
Probability
1.0
0.5
0.0
1
2
3
4
5
6
7
8
9
10
11
d
R=0.9993, P = 1.563e-183
R=0.9983, P = e-5306
AGA codon_Enriched protein
AGA codon_Unriched protein
log2 (gctrl−Abundance)
log2 (RESTART v3−Abundance)
e
Density
0.6
0.4
0.2
0
5'UTR
CDS
3'UTR
f
Otof c.1315 C>T
Otof c.1315 C>T + AAVie-RESTART v3
Modification level (%)
XM_006539713.5
NM_001290367.1
NM_139269.2
NM_133898.4
NM_001113379.2
NM_172924.3
XM_006507979.4
XM_036155008.1
XR_003955567.2
NM_001363323.1
XM_006535084.4
NR_156476.1
NM_153164.4
NM_001402879.1
NM_133969.3
NM_138679.5
NM_001289740.1
NM_008139.6
NM_001080977.1
NM_001033430.4
NM_001403458.1
NM_001411829.1
NM_001310629.1
NM_177445.5
NM_010595.3
NM_025867.2
NM_011127.2
NM_018823.2
NM_175489.3
NM_001081336.2
NM_001347176.2
NM_145226.2
NM_007620.3
NM_011682.4
NM_207633.2
NM_010864.2
NM_001081145.1
NM_153404.3
NM_001204983.2
NM_001402823.1
NM_031494.2
NM_019400.3
NM_013510.4
NM_001311161.1
NM_001018042.3
NM_146185.2
NM_001368755.1
XM_006530870.2
XM_006507187.5
XM_006501142.3

Supplementary Figure 6 | Off-target analysis of RESTART v3.

(a) qRT-PCR results showing relative *Otof* expression levels in WT and *Otof c.1315 C>T* mice with or without RESTART v3 treatment. n = 4 biological samples. The statistical analyses are unpaired Student’s t tests. Data are represented as the mean ± SD. **(b)** Pie charts show the proportion of off-target Ψ sites in the 5′UTR, 3′UTR, and CDS regions in *in vitro* cell line experiments. **(c)** Sequence motif of the identified off-target Ψ sites in *in vitro* cell line experiments. **(d)** Scatterplot shows the expression level of AGA codon enriched proteins by quantitative proteome analysis. Cells were transfected with the RESTART v3 or gctrl (only transfect with non-targeting control gsnoRNA). The two-sided Pearson correlation test performs correlation analysis based on the $\log_2$-transformed mean abundance values detected in replicates of the gctrl group and the RESTART v3 treatment group. R represents the Pearson correlation coefficient, and the $p < 0.0001$ indicates a significant correlation between the two groups. **(e)** Metagene profiles showing the distribution of identified endogenous Ψ sites. Each segment is normalized according to its average length in RefSeq annotation. **(f)** The Ψ modification status in predicted the top 50 regions resembling gACA19 complementary sequences.

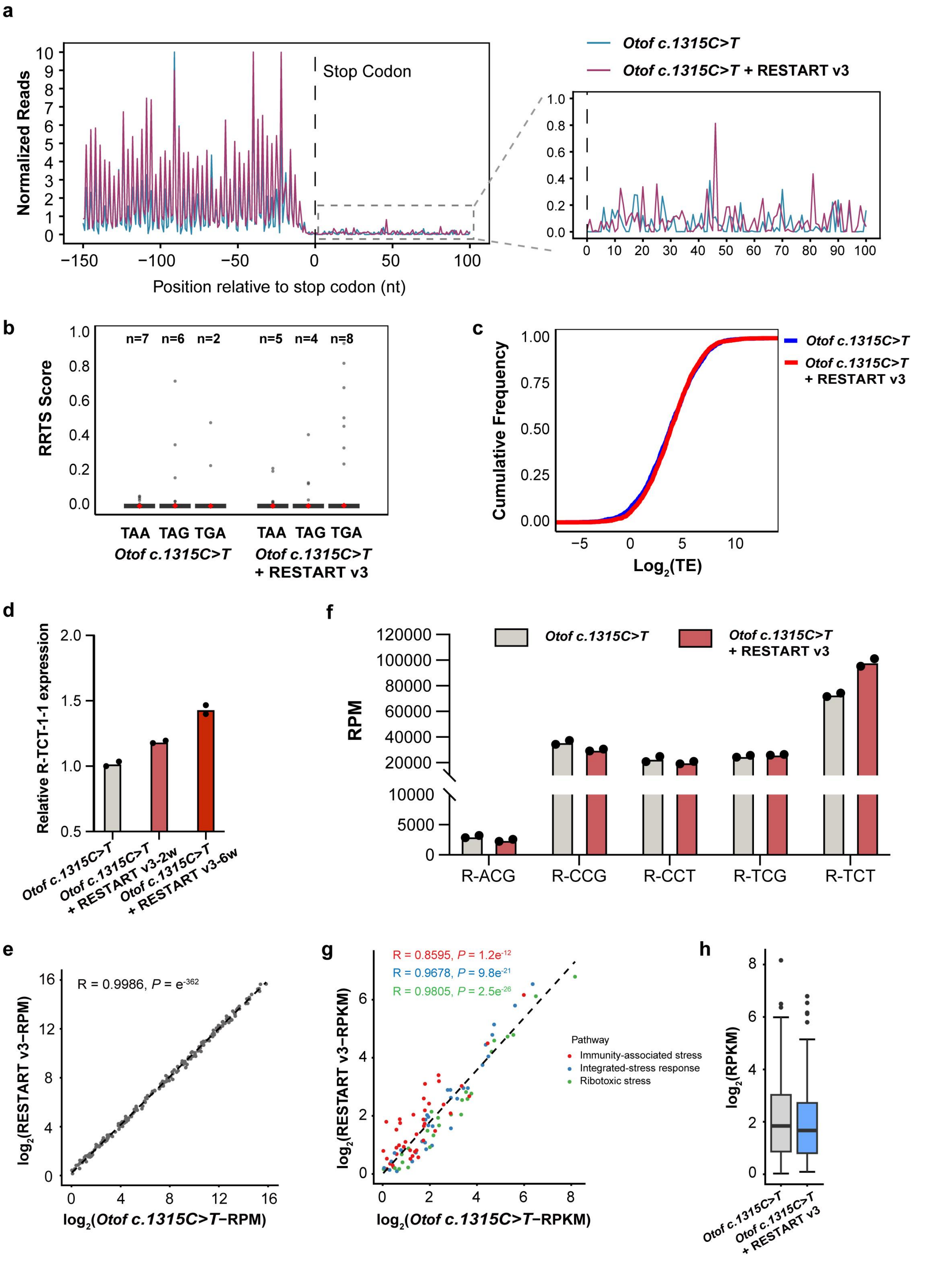
a
Normalized Reads
Stop Codon
Position relative to stop codon (nt)
Otof c.1315C>T
Otof c.1315C>T + RESTART v3
b
RRTS Score
n=7 n=6 n=2 n=5 n=4 n=8
TAA TAG TGA TAA TAG TGA
Otof c.1315C>T
Otof c.1315C>T + RESTART v3
c
Cumulative Frequency
Log2(TE)
Otof c.1315C>T
Otof c.1315C>T + RESTART v3
d
Relative R-TCT-1-1 expression
Otof c.1315C>T
Otof c.1315C>T + RESTART v3-2w
Otof c.1315C>T + RESTART v3-6w
f
RPM
Otof c.1315C>T
Otof c.1315C>T + RESTART v3
R-ACG R-CCG R-CCT R-TCG R-TCT
e
R = 0.9986, P = e-362
log2(RESTART v3−RPM)
log2(Otof c.1315C>T−RPM)
g
R = 0.8595, P = 1.2e-12
R = 0.9678, P = 9.8e-21
R = 0.9805, P = 2.5e-26
Pathway
Immunity-associated stress
Integrated-stress response
Ribotoxic stress
log2(RESTART v3−RPKM)
log2(Otof c.1315C>T−RPKM)
h
log2(RPKM)
Otof c.1315C>T
Otof c.1315C>T + RESTART v3

Supplementary Figure 7 | Specificity and molecular safety analysis of RESTART v3.

(a) Metagene plot showing normalized reads of ribosome-protected fragments (RPFs) relative to the distance from the normal stop codon at position 0. **(b)** Box plot of ribosome readthrough score (RRTS) values derived from ribosome profiling of mice cochlear tissues with or without RESTART v3 treatment. RRTS values were calculated for transcripts harboring different normal stop codons, UAA, UAG and UGA. The center line indicates the median, the box ends indicate the first and third quartiles. n = 3 biological replicates. **(c)** Cumulative distribution of protein-coding gene translation efficiency in *Otof c.1315 C>T* mice with and without RESTART v3 treatment. **(d)** Expression level of tRNA-R-TCT-1-1 in the mouse cochlea before, 2 weeks (2w) after, and 6 weeks (6w) after RESTART v3 treatment. n = 2 biological replicates. **(e)** Differential expression analysis of tRNA transcripts grouped by iso-decoders in AAVie RESTART v3-treated cochlea relative to untreated samples. Data are represented as the mean value of two biological replicates. Correlation analysis is performed by the two-sided Pearson correlation test based on the $\log_2$-transformed mean RPM values detected in replicates of the *Otof c.1315 C>T* mice with and without RESTART v3 treatment. R represents the Pearson correlation coefficient, and the $p < 0.0001$ indicates a significant correlation between the two groups. **(f)** Expression levels of the Arg-isoacceptor family in mouse cochlear tissue, untreated and after 6 weeks of RESTART v3 treatment. n = 2 biological replicates. **(g)** A scatter plot from transcriptome displaying the expression levels of stress-related genes in mouse cochlear with and without RESTART v3 treatment. The two-sided Pearson correlation test performs correlation analysis based on the $\log_2$-transformed mean RPKM values detected in replicates of the *Otof c.1315 C>T* mice with and without RESTART v3 treatment. **(h)** Box plot displaying the expression levels of stress-related genes in mouse cochlear with and without RESTART v3 treatment. The center line indicates the median, the box ends indicate the first and third quartiles.

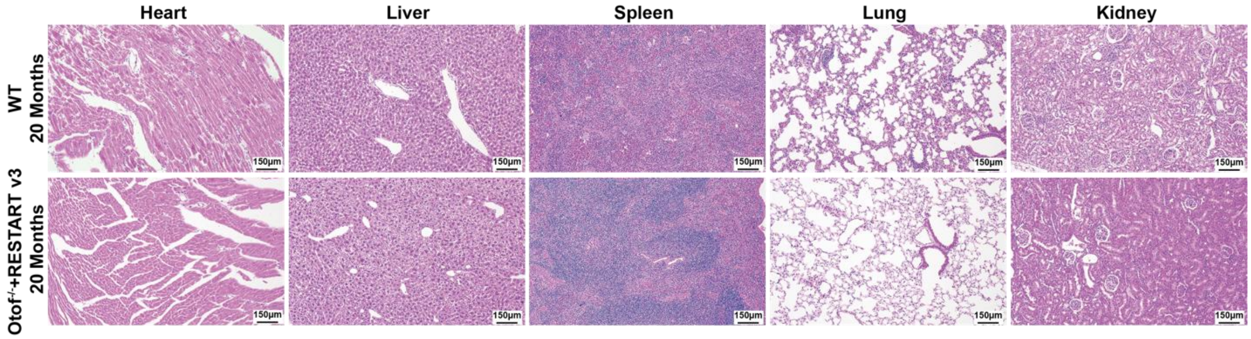

Supplementary Figure 8 | Long-term safety evaluation of RESTART v3 – AAV system.

HE staining showed no significant differences in organ tissue structures between WT and AAVie-RESTART v3 treated *Otof c.1315C>T* (p.R439*) mice (~19 months post - administration). Scale Bars, 150 μm. The experiment was performed in three independent replicates, all of which yielded consistent results.

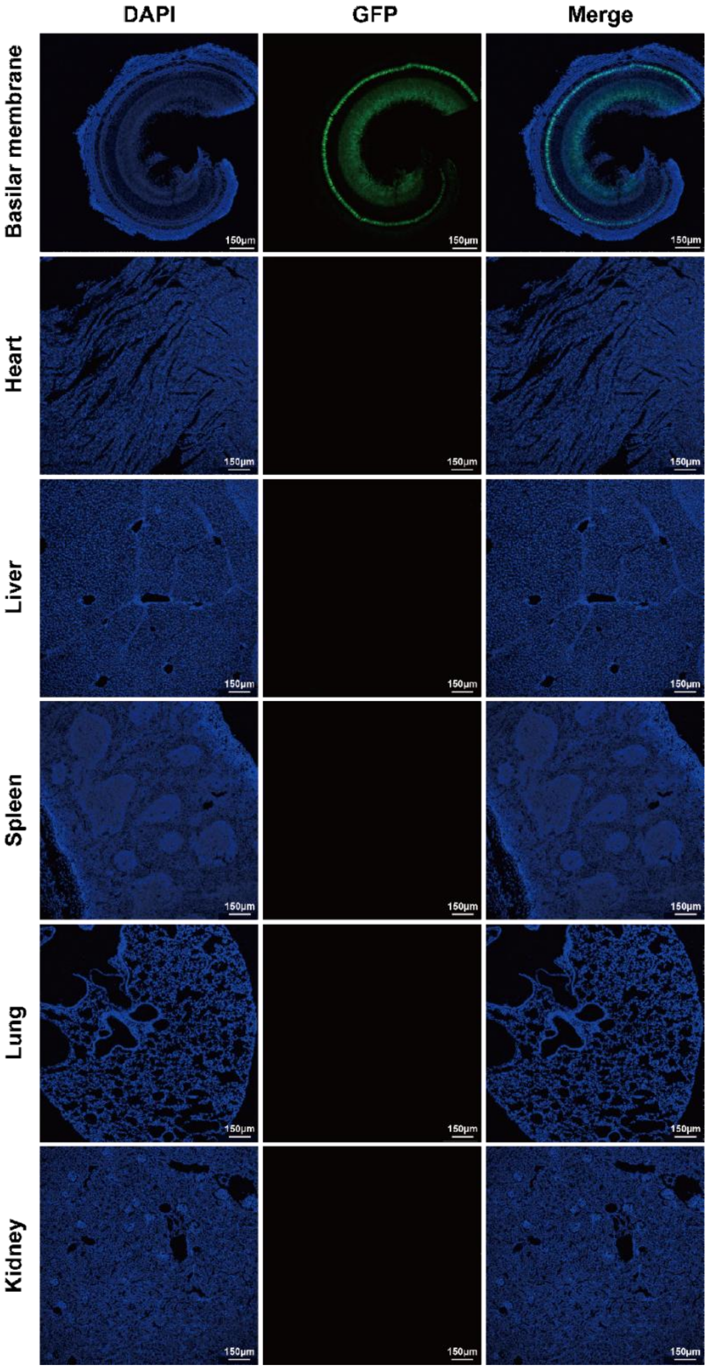

Supplementary Figure 9 | The tissue-specific expression of the AAVie deliver system. Administration of the AAVie-vector resulted in robust transgene expression (green) exclusively within the cochlea, with no detectable signal observed in other major organs (e.g., heart, liver, spleen, lung, and kidney). This confirms the highly specific targeting capability of the system for inner ear. Scale Bars, 150 μm. Consistent results were obtained across all three experimental replicates.

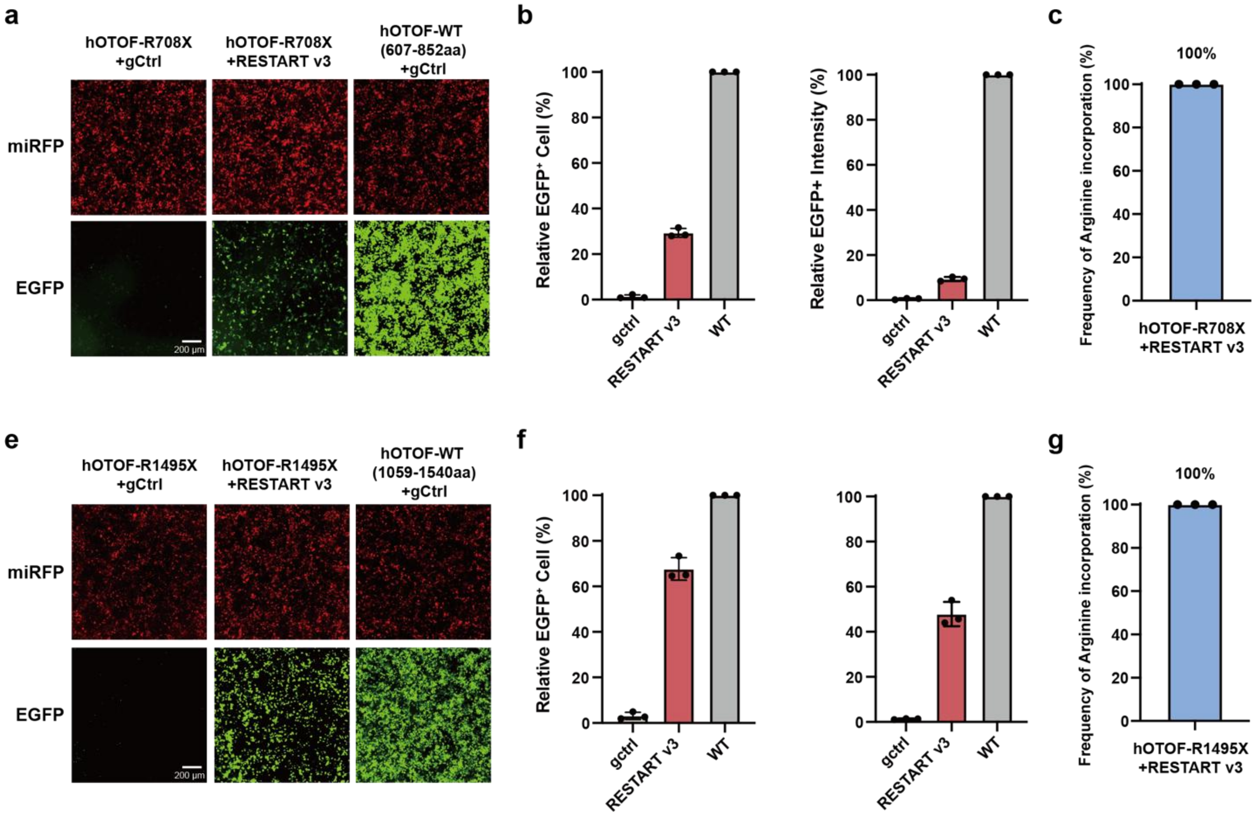

Supplementary Figure 10 | RESTART v3 corrects other human-deafness-associated nonsense mutations within *OTOF* gene. (a-c) The hOTOF R708X-reporter and RESTART v3 were transfected into HEK293T cells. Representative fluorescence images of cells. The experiment was repeated three times, and yielded consistent results across all replicates **(a)**. Bar plots showing the relative fraction of EGFP positive cells and intensity **(b)**. Bar plot showing the arginine incorporation rate at human *OTOF* (p. R708X) PTC site **(c)**. **(e-g)** The hOTOF R1495X-reporter and RESTART v3 were transfected into HEK293T cells. Representative fluorescence images of cells. The experiment was conducted in three biological replicates with consistent results. **(e)**. Bar plots showing the relative fraction of EGFP positive cells and intensity **(f)**. Bar plot showing the arginine incorporation rate at human *OTOF* (p. R1495X) PTC site **(g)**. **(a-g)** n = 3 biological replicates. Data are represented as the mean ± SD. Scale Bars, 200 μm.
