# Supplementary material for: CRISPR-free RNA base editing mediated PTC-readthrough restores hearing in mice with Otof nonsense mutation
Source: Nat Commun. 2025 Dec 6;17:413. doi: 10.1038/s41467-025-67112-w (PMC12796263; doi:10.1038/s41467-025-67112-w)
Supplement: Supplementary file 2 — Description of Additional Supplementary Files [file 41467_2025_67112_MOESM2_ESM.pdf]

Description of Additional Supplementary Files

File Name: Supplementary Data 1
Description: The identified endogenous Ψ modification sites under in vivo condition.

File Name: Supplementary Data 2
Description: The Ψ modification status in predicted the all regions resembling guide snoRNA complementary sequences.

File Name: Supplementary Data 3
Description: Complete blood count (CBC) tests among the WT, AAVie-GFP and AAVie-RESTART v3 treated mice.

File Name: Supplementary Data 4
Description: Serum biochemical detection among the WT, AAVie-GFP and AAVie-RESTART v3 treated mice.

File Name: Supplementary Data 5
Description: The sequences of guide snoRNAs and primers.

File Name: Supplementary Movie 1
Description: Auditory startle reflex of treated *Otof c.1315C>T* (p.R439X) mice.

File Name: Supplementary Movie 2
Description: Auditory startle reflex elicited by clap in treated *Otof c.1315C>T* (p.R439X) mice.
