# Supplementary material for: CRISPR-free RNA base editing mediated PTC-readthrough restores hearing in mice with Otof nonsense mutation
Source: Nat Commun. 2025 Dec 6;17:413. doi: 10.1038/s41467-025-67112-w (PMC12796263; doi:10.1038/s41467-025-67112-w)
Supplement: Supplementary file 10 — Reporting Summary [file 41467_2025_67112_MOESM10_ESM.pdf]

nature portfolio

Corresponding author(s): Ke Liu

Last updated by author(s): Nov 12, 2025

Reporting Summary

Nature Portfolio wishes to improve the reproducibility of the work that we publish. This form provides structure for consistency and transparency in reporting. For further information on Nature Portfolio policies, see our Editorial Policies and the Editorial Policy Checklist.

Statistics

For all statistical analyses, confirm that the following items are present in the figure legend, table legend, main text, or Methods section.

n/a	Confirmed	
☐	☒	The exact sample size (*n*) for each experimental group/condition, given as a discrete number and unit of measurement
☐	☒	A statement on whether measurements were taken from distinct samples or whether the same sample was measured repeatedly
☐	☒	The statistical test(s) used AND whether they are one- or two-sided<br>*Only common tests should be described solely by name; describe more complex techniques in the Methods section.*
☒	☐	A description of all covariates tested
☒	☐	A description of any assumptions or corrections, such as tests of normality and adjustment for multiple comparisons
☐	☒	A full description of the statistical parameters including central tendency (e.g. means) or other basic estimates (e.g. regression coefficient) AND variation (e.g. standard deviation) or associated estimates of uncertainty (e.g. confidence intervals)
☐	☒	For null hypothesis testing, the test statistic (e.g. *F*, *t*, *r*) with confidence intervals, effect sizes, degrees of freedom and *P* value noted<br>*Give P values as exact values whenever suitable.*
☒	☐	For Bayesian analysis, information on the choice of priors and Markov chain Monte Carlo settings
☒	☐	For hierarchical and complex designs, identification of the appropriate level for tests and full reporting of outcomes
☐	☒	Estimates of effect sizes (e.g. Cohen's *d*, Pearson's *r*), indicating how they were calculated

Our web collection on statistics for biologists contains articles on many of the points above.

Software and code

Policy information about availability of computer code

Data collection	Next-generation sequencing data was observed and demultiplexed by Illumina NovaSeq platforms and BGI platforms. Cells were imaged using the ImageXpress Micro 4 high-content imaging system. Audiological data of mice were collected by the SigGen/BioSig software. Images of the basilar membrane of the cochlea were acquired by TCS SP8 II Leica. ASR related videos were obtained through capcut v5.5.0.

Data analysis

Only read 2 was used for subsequent analysis.
The adapter sequences were removed in raw reads with cutadapt software (v4.2).
Duplicated reads were removed by Seqkit (v0.13.2).
UMI were removed by umi_tools (v1.0.0).
Cleaned genes reads were aligned to the corresponding amplicon reference with STAR algorithm (v2.7.10b).
The genes counts were quantified by HTSeq(v1.14), and genes with low biological repeatability (log2 fold change(replication 1/replication 2)>1) were discarded.
The genes expression level was quantified by RPKM.
Target amplicon sequencing data was analyzed using the published toolkit PRAISE (v1.0.0) (https://github.com/Zhe-jiang/PRAISE).
PRAISE sequencing data was analyzed using the published tookit PRAISE (v1.0.0) (https://github.com/Zhe-jiang/PRAISE).
Off-target sites were detected using suitable cutoffs.
On-target sites were detected using bowtie(v1.3.1) with suitable cutoffs.
The cleaned reads from tRNA-seq and Ribo-seqwere mapped to the reference using bowtie2 (version 2.5.4) and STAR algorithm (v2.7.10b).
The mapped alignments were processed by samtools (version 1.14).
The tRNA counts were quantified by pysam (version 0.23.3).
P-site positions within ribosome-protected fragments were precisely determined using the R package riboWaltz.
GraphPad Prism 9 was used for basic statistical analysis and graph production.

For manuscripts utilizing custom algorithms or software that are central to the research but not yet described in published literature, software must be made available to editors and reviewers. We strongly encourage code deposition in a community repository (e.g. GitHub). See the Nature Portfolio guidelines for submitting code & software for further information.

Data

Policy information about availability of data

All manuscripts must include a data availability statement. This statement should provide the following information, where applicable:

- Accession codes, unique identifiers, or web links for publicly available datasets
- A description of any restrictions on data availability
- For clinical datasets or third party data, please ensure that the statement adheres to our policy

The sequence data generated in this study have been deposited in the NCBI Gene Expression Omnibus (GEO) and Genome Sequence Archive (GSA), under accession code GSE262204 [https://www.ncbi.nlm.nih.gov/geo/query/acc.cgi?acc=GSE262204], GSE262205 [https://www.ncbi.nlm.nih.gov/geo/query/acc.cgi?acc=GSE262205], GSE262206 [https://www.ncbi.nlm.nih.gov/geo/query/acc.cgi?acc=GSE262206], PRJCA049346 [https://ngdc.cncb.ac.cn/bioproject/browse/PRJCA049346], and PRJCA049089 [https://ngdc.cncb.ac.cn/bioproject/browse/PRJCA049089]. The mass spectrometry proteomics data have been deposited to the ProteomeXchange Consortium via the iProX partner repository with the dataset identifier PXD070139 [https://proteomecentral.proteomexchange.org/cgi/GetDataset?ID=PXD070139]. Source data are provided with this paper.

Research involving human participants, their data, or biological material

Policy information about studies with human participants or human data. See also policy information about sex, gender (identity/presentation), and sexual orientation and race, ethnicity and racism.

Reporting on sex and gender	N/A
Reporting on race, ethnicity, or other socially relevant groupings	N/A
Population characteristics	N/A
Recruitment	N/A
Ethics oversight	N/A

Note that full information on the approval of the study protocol must also be provided in the manuscript.

Field-specific reporting

Please select the one below that is the best fit for your research. If you are not sure, read the appropriate sections before making your selection.

☒ Life sciences ☐ Behavioural & social sciences ☐ Ecological, evolutionary & environmental sciences

For a reference copy of the document with all sections, see nature.com/documents/nr-reporting-summary-flat.pdf

Life sciences study design

All studies must disclose on these points even when the disclosure is negative.

Sample size

No statistical methods were used to predetermine sample size. Target-seq of reporter transcripts or endogenous transcripts was done with independent experiments performed in parallel. The minimum 4 biological replicates were performed to confirm reproducibility. Two

independent transcriptome-wide RNA seq and tRNA seq were performed. The sample size was determined based on published paper (Luo, et. al, Nat. Biotechnol., 2025, PMID: 38448662). Three independent ribo-seq were performed. For experiments on auditory phenotypes in mice, a minimum of 3 biological replicates were performed to confirm reproducibility, and the number of replicates was listed in the text or figure legends.

Data exclusions	No data was excluded.
Replication	All experiments were carried out in at least 2 biological replicates. All attempts to replicate data were successful.
Randomization	Samples were randomly allocated into experimental groups.
Blinding	No blinding was performed due to the involvement of several experimenters.

Reporting for specific materials, systems and methods

We require information from authors about some types of materials, experimental systems and methods used in many studies. Here, indicate whether each material, system or method listed is relevant to your study. If you are not sure if a list item applies to your research, read the appropriate section before selecting a response.

Materials & experimental systems

n/a	Involved in the study	
☐	☒	Antibodies
☐	☒	Eukaryotic cell lines
☒	☐	Palaeontology and archaeology
☐	☒	Animals and other organisms
☒	☐	Clinical data
☒	☐	Dual use research of concern
☒	☐	Plants

Methods

n/a	Involved in the study	
☒	☐	ChIP-seq
☒	☐	Flow cytometry
☒	☐	MRI-based neuroimaging

Antibodies

Antibodies used	Anti-otoferlin antibody, Rabbit (ab309197, 1:300, Abcam) ;<br>Anti-myosin VIIa antibody, Mouse (25–6790, 1:300, Proteus BioSciences);<br>Alexa FluorTM 594 Goat anti-mouse (1:300, Invitrogen, A11005);<br>Alexa FluorTM 488 Goat anti-rabbit,(1:300, Invitrogen, A-11008)
Validation	All antibodies used in this study were validated by the manufacturer.<br>1) anti-otoferlin antibody (validated by the manufacturer by immunohistochemical analysis of 4% PFA-fixed, 0.2% Triton X-100 permeabilized frozen Rat cochlea tissue; validation provided on website: https://www.abcam.cn/products/primary-antibodies/otoferlin-antibody-epr28223-1-ab309197);<br>2) anti-myosin VIIa antibody (validation provided on website: https://www.proteus-biosciences.com/search/25-6790);<br>3) Secondary antibody, Alexa FluorTM 594 Goat anti-mouse (cited in 2746 publication; validated by the manufacturer by immunohistochemical analysis of Human iPSC Staining Tubulin ; validation provided on website: https://www.thermofisher.cn/cn/zh/antibody/product/Goat-anti-Mouse-IgG-H-L-Cross-Adsorbed-Secondary-Antibody-Polyclonal/A-11005);<br>4) Secondary antibody, Alexa FluorTM 488 Goat anti-rabbit (cited in 11504 publication; validated by the manufacturer by immunohistochemical analysis of HeLa cells stained with alpha Tubulin Rabbit Polyclonal Antibody; validation provided on website: https://www.thermofisher.cn/cn/zh/antibody/product/Goat-anti-Rabbit-IgG-H-L-Cross-Adsorbed-Secondary-Antibody-Polyclonal/A-11008)

Eukaryotic cell lines

Policy information about cell lines and Sex and Gender in Research

Cell line source(s)	HEK293T cells were purchased from ATCC (ATCC CRL-3216).
Authentication	No method of cell line authentication was used.
Mycoplasma contamination	Cells were tested negative for mycoplasma contamination.
Commonly misidentified lines<br>(See ICLAC register)	No commonly misidentified lines were used.

Animals and other research organisms

Policy information about studies involving animals; ARRIVE guidelines recommended for reporting animal research, and Sex and Gender in Research

Laboratory animals	Four weeks old otof c.1315C>T (p.R439*) mice used as model animals. C57BL/6J mice used as control group.
Wild animals	This study did not involve wild animals.
Reporting on sex	Animals were randomly allocated to experimental groups without regard to sex, as it is not a known confounding variable for the endpoints being measured.
Field-collected samples	All the mice were bred and housed in the Experimental Animal Department of the Capital Medical University (22-25 ∘C, 50% humidity, 12 h light/dark cycle).
Ethics oversight	All experiments were approved by the Animal Ethics Committee of Capital Medical University

Note that full information on the approval of the study protocol must also be provided in the manuscript.

Plants

Seed stocks	N/A
Novel plant genotypes	N/A
Authentication	N/A
